# Hallazgos clínicos, radiológicos y moleculares de la omodisplasia de tipo 2

**DOI:** 10.7705/biomedica.7922

**Published:** 2026-10-16

**Authors:** Francisco Cammarata-Scalisi, Antonio Cárdenas-Tadich, Carmen Ludeña, Maykol Araya-Castillo, Michele Callea

**Affiliations:** 1 Servicio de Pediatría, Hospital Regional de Antofagasta, Antofagasta, Chile Hospital Regional de Antofagasta Hospital Regional de Antofagasta Antofagasta Chile; 2 Laboratorio Clínico, Hospital Regional de Antofagasta, Antofagasta, Chile Hospital Regional de Antofagasta Hospital Regional de Antofagasta Antofagasta Chile; 3 Programa de Cirugía Oral, Universidad de Florencia, Florencia, Italia Universidad de Florencia Universidad de Florencia Florencia Italia; 4 Doctoral Study Programme Molecular Genetics and Biotechnology, University of Nova Gorica, Nova Gorica, Slovenia University of Nova Gorica University of Nova Gorica Nova Gorica Slovenia

**Keywords:** trastornos del crecimiento, osteocondrodisplasias/genética, diagnóstico por imagen, técnicas de diagnóstico molecular, asesoramiento genético, Growth disorders, osteochondrodysplasias/genetics, diagnostic imaging, omodysplasia, clinic, diagnostic by image, molecular diagnostic techniques, genetic counselling

## Abstract

La omodisplasia es un trastorno esquelético congénito y raro. Presenta un patrón de herencia autosómico recesivo denominado omodisplasia de tipo 1 y otro, autosómico dominante conocido como omodisplasia de tipo 2, el cual se caracteriza clínicamente por acortamiento rizomélico con predominio en las extremidades superiores, dislocación de la cabeza radial, primeros metacarpianos cortos, dismorfia facial y anomalías genitourinarias. Las variantes en heterocigosis del gen *FZD2* se identifican como la causa de la omodisplasia de tipo 2, que codifica a la proteína Frizzled-2.

Se presenta el caso de un adolescente de sexo masculino, quien presenta hallazgos clínicos y radiológicos de omodisplasia de tipo 2, con labio hendido bilateral, acortamiento rizomélico con predominio en los miembros superiores, acortamiento del primer metacarpiano, micropene y testículo en ascensor. El estudio de secuenciación del exoma arrojó variante sin sentido, probablemente patogénica, en heterocigosis en el gen *FZD2* c.1640C>A p.(Ser547*). La confirmación del diagnóstico sindromático permitió brindar una evaluación médica multidisciplinaria y brindar un oportuno asesoramiento genético familiar.

## Abstract (en)

Omodysplasia is a rare congenital skeletal disorder. It has an autosomal recessive inheritance pattern known as omodysplasia 1 and an autosomal dominant pattern known as omodysplasia 2, which is clinically characterized by predominantly rhizomelic shortening of the upper limbs, radial head dislocation, short first metacarpals, facial dysmorphism, and genitourinary anomalies. Heterozygous variants in the *FZD2* gene have been identified as the cause of omodysplasia 2, which encodes the Frizzled-2 protein.

A male adolescent is presented with clinical and radiological findings of omodysplasia 2: bilateral cleft lip, rhizomelic shortening predominantly in the upper limbs, shortening of the first metacarpal, micropenis, and retractile testicle. Exome sequencing revealed a likely pathogenic heterozygous missense variant in the *FZD2* gene c.1640C>A p.(Ser547*). Confirming the syndromic diagnosis allows for a multidisciplinary medical evaluation and timely family genetic counseling.

La omodisplasia es un trastorno esquelético congénito y raro ^1^^-^^5^, que fue diferenciado clínicamente en 1989 por Maroteaux *et al*. ^5^. Este trastorno presenta dos patrones: el primero, de herencia autosómica recesiva, denominado omodisplasia de tipo 1 (OMOD1; OMIM #258315), es causado por variantes en homocigosis o heterocigosis compuesta del gen *GPC6* (OMIM *604404) ^6^; el segundo, de herencia autosómica dominante, conocido como omodisplasia de tipo 2 (OMOD2; OMIM #164745) ^1^^,^^2^^,^^6^, es causado por variantes en heterocigosis del gen *FZD2* (OMIM *600667) ^6^, que resalta por su heterogeneidad genética ^2^^,^^3^. Ambos tipos se encuentran en el grupo 17 de las displasias mesomélicas y rizomesomélicas, según la nosología de los trastornos esqueléticos ^7^.

La omodisplasia de tipo 2 se caracteriza clínicamente por el acortamiento de las extremidades superiores con predominio rizomélico, dislocación de la cabeza radial ^1^^,^^8^, primeros metacarpianos cortos (rasgo distintivo)^1^^,^^6^^,^^8^, dismorfia facial (cara redondeada, prominencia frontal, nariz corta y surco nasolabial largo), y anomalías genitourinarias ^1^^,^^8^. De forma variable, pueden cursar con baja estatura, anomalías vertebrales y femorales ^1^.

Las variantes en heterocigosis del gen *FZD2*, localizado en 17q21.31, se identifican como la causa de la omodisplasia de tipo 2, que codifica a la proteína Frizzled-2 ^2^^,^^6^, la cual actúa como una vía de señalización Wnt y se expresa durante el desarrollo facial y esquelético en múltiples sistemas de modelos ^2^^,^^6^^,^^8^.

Se presenta un adolescente de sexo masculino, quien presenta hallazgos clínicos y radiológicos de omodisplasia de tipo 2 -confirmada por estudio genético molecular-, con el objetivo de recibir una evaluación médica multidisciplinaria y un oportuno asesoramiento genético familiar.

## Presentación del caso

Se trata de un paciente de sexo masculino, de 11 años, el tercero de una prole de cuatro hermanos, caso único en la familia y producto de padres no consanguíneos, procedentes de Bolivia. Su gestación fue controlada; nació a las 39 semanas, con un peso de 3100 g [mediana - (-1DE)] y talla de 48 cm (-1DE), sin diagnóstico prenatal. Al momento de nacer, se describió labio hendido bilateral y acortamiento rizomélico con predominio en los miembros superiores.

El desarrollo psicomotor y el desempeño escolar del paciente fueron adecuados. Fue intervenido quirúrgicamente a los 6 meses y reintervenido a los 7 años debido a su labio hendido. También, fue hospitalizado al año de edad por infección de las vías urinarias, sin complicaciones. Además, se sometió a una hernioplastia umbilical a los 8 años.

En el examen físico, se identificó prominencia frontal, cejas anchas y escasas, ptosis palpebral, puente nasal ancho, narinas antevertidas, columela corta, cicatriz a nivel del *filtrum* por el antecedente de labio hendido, pabellones auriculares de baja implantación, rotadas posteriormente y en asa. Además, agenesia y persistencia de algunas piezas dentales, *pectus excavatum* y micropene con antecedentes de testículo en ascensor. A nivel de las extremidades, se encontró acortamiento rizomélico.

Las medidas de antropometría y de los diferentes segmentos corporales se describen en el cuadro 1. Los estudios de radiología simple de miembros superiores evidenciaron acortamiento del húmero, alteración del radio y acortamiento del primer metacarpiano (figura 1).

**Cuadro 1:** Medidas antropométricas y de los segmentos corporales del paciente estudiado

Antropometría	Medida	Desviación estándar (DE)
Talla (cm)	132	+1 a -2 DE
Peso (kg)	27,8	Mediana a -1 DE
		Percentil
Brazo (cm)	16,1	< 5
Antebrazo (cm)	17,1	< 5
Mano (cm)	14,5	< 3
Muslo (cm)	32	< 3
Pierna (cm)	29	< 5
Pie (cm)	21	25-3

**Figura 1:**
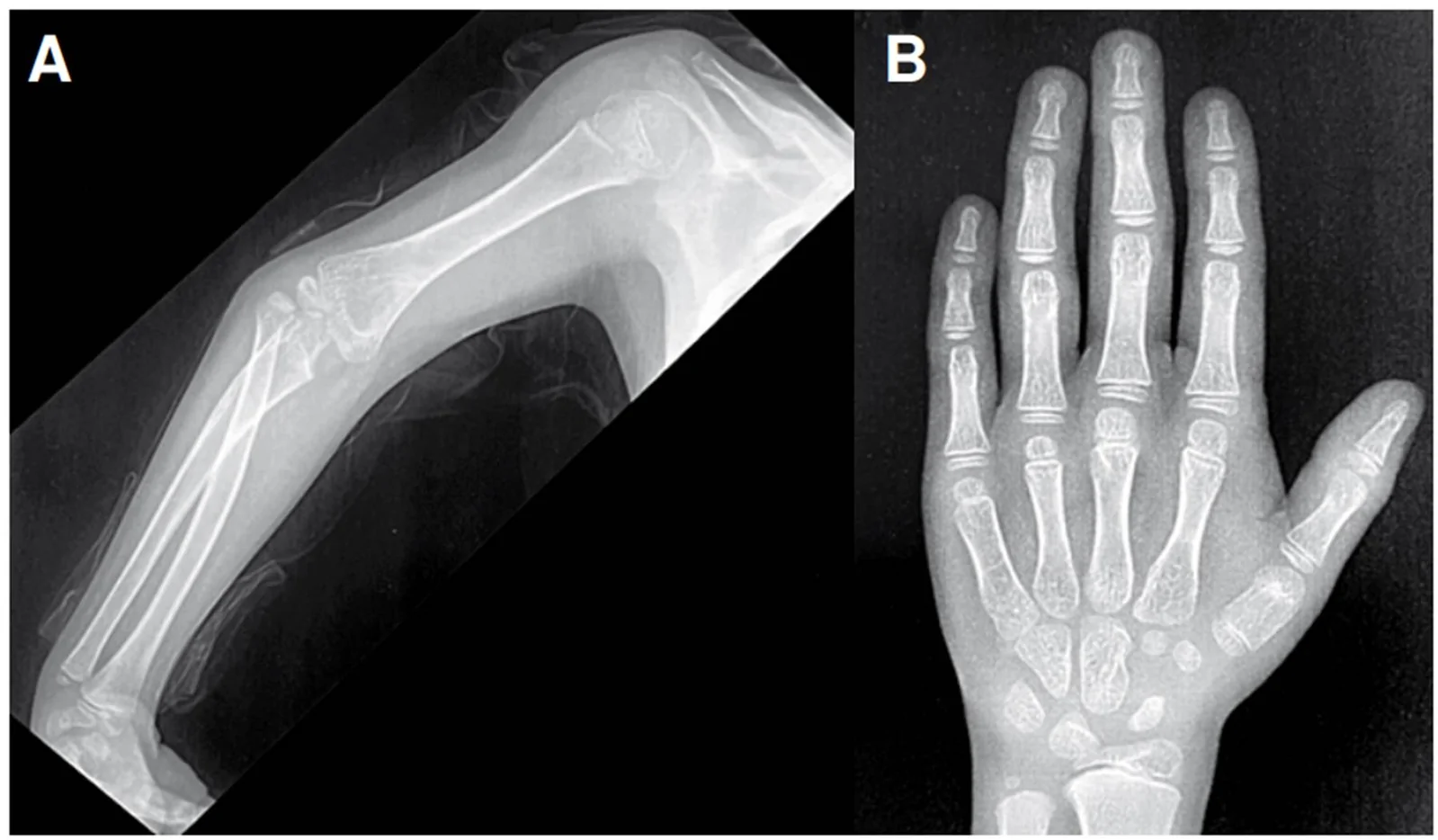
A. Radiografía de miembro superior en la que se observa acortamiento del húmero y alteración del radio. B. Acortamiento de primer metacarpiano.

El estudio de secuenciación de exoma arrojó como resultado una variante sin sentido, probablemente patogénica, en heterocigosis del gen *FZD2* c.1640C>A p.(Ser547*).

### Consideraciones éticas

Este estudio fue autorizado por la dirección del Hospital Regional de Antofagasta. Se obtuvo el consentimiento informado de la madre del paciente para la realización del estudio genético, la discusión del caso con especialistas expertos en el área de displasias esqueléticas, y su presentación en jornadas y revistas científicas.

## Discusión

La omodisplasia es un grupo heterogéneo de displasias óseas micromélicas ^6^. Hasta la fecha, solo se han documentado 13 casos de omodisplasia de tipo 2 en la literatura ^1^^,^^6^^,^^8^^,^^9^ y este es el primer caso reportado en Latinoamérica. En el cuadro 2, se describen los hallazgos clínicos de este caso clínico en comparación con los anteriormente reportados. La variante descrita en este informe fue reportada en un paciente de sexo masculino por Nagasaki *et al*. ^6^, en Japón. Además de esta, se han descrito otras dos variantes en cinco pacientes: c.1301G>T (p.Gln434Val) ^1^^,^^2^, y c.1644G>A (p.Trp548*) ^1^^,^^8^, lo que sugiere que estas variantes pueden ser recurrentes ^1^.

**Cuadro 2:** Hallazgos clínicos del presente paciente y del estudiado por Nagasaki *et al*. (con la misma variante génica) y la frecuencia de los casos documentados en la literatura ^1^^-^^6^^,^^8^

Hallazgos		Presente caso	**Nagasaki *et al*.**	Frecuencia
Sexo				
	Femenino			8/13
	Masculino	+	+	5/13
Talla baja		-	+	5/12
Hallazgos faciales				
	Facies redondeada	-	-	8/13
	Región frontal prominente	+	-	9/12
	Hipertelorismo	+	+	7/11
	Puente nasal ancho	+	-	11/13
	Nariz pequeña con punta ancha	+	+	12/13
	Filtrum largo	-	+	7/13
	Microrretrognatia	-	-	5/13
	Labio y paladar hendidos	+	-	3/13
Hallazgos esqueléticos				
	Húmero corto	+	+	13/13
	Dislocación radial	+	+	13/13
	Limitación de la articulación del codo	+	+	9/11
	Cúbito corto	+	-	10/13
	Primeros huesos metacarpianos cortos	+	+	12/13
	Peroné corto	-	-	2/11
Alteración genital		+	+	7/11

+: presente; -: ausente

La variante descrita en el paciente (c.1640C>A p.(Ser547*)) y la p.Trp548* predicen la pérdida de la cola citoplasmática intracelular, responsable de la interacción canónica de Dishevelled ^6^. La forma truncada de la proteína conduce al retraso o bloqueo de la maduración de los condrocitos y al acortamiento de los elementos esqueléticos ^8^.

El receptor anormal interfiere con la vía de señalización canónica de Wnt, que desempeña un papel fundamental durante la embriogénesis y el desarrollo. La unión de los ligandos de Wnt a los complejos receptores activa las vías independientes de señalización canónicas y no canónicas de la β-catenina.

La señalización anormal de Wnt -particularmente la inducida por Wnt5a- es responsable, no solo de la omodisplasia de tipo 2, sino también, de la omodisplasia de tipo 1 y del síndrome de Robinow. Por lo tanto, el deterioro de la señalización de Wnt dificulta el desarrollo normal de muchos órganos y tejidos, lo que da como resultado el acortamiento de las extremidades, la dismorfia facial y la hipoplasia genital ^6^.

La omodisplasia de tipo 2 se asemeja al síndrome de Robinow autosómico dominante (SRAD; OMIM #180700). Por un lado, en la omodisplasia de tipo 2 se puede presentar estatura normal ^10^, importante acortamiento rizomélico de las extremidades ^6^, primeros metacarpianos acortados, húmeros acortados con cóndilos hipoplásicos ^10^, alteraciones dentales y genitales masculinos hipoplásicos, como micropene, hipospadias y criptorquidia. En el rostro, se presenta prominencia frontal ^6^^,^^10^, nariz corta ^6^, puente nasal deprimido con punta bífida y surco nasolabial largo ^6^^,^^10^. El dimorfismo facial se presenta de manera más leve en la omodisplasia de tipo 2 que en el síndrome de Robinow autosómico ^6^, lo que constituye el principal diagnóstico diferencial. Ambas enfermedades se encuentran en el grupo 17 de las displasias mesomélicas y rizomesomélicas, según la nosología de los trastornos esqueléticos ^7^.

Por otro lado, el síndrome de Robinow autosómico se caracteriza por baja estatura, acortamiento mesomélico de las extremidades superiores de forma predominante ^2^^,^^8^^,^^10^ y braquidactilia. Los rasgos faciales se caracterizan por prominencia frontal ^10^, hipertelorismo con ojos prominentes ^2^^,^^8^^,^^10^ e inclinados hacia arriba, nariz pequeña, puente nasal ancho ^2^, narinas antevertidas, retrusión del tercio medio facial, anomalías dentales ^10^ e hipoplasia mandibular ^8^.

Los pacientes con este síndrome presentan, ocasionalmente, macrocefalia prenatal que persiste después del nacimiento ^10^. Entre las anomalías genitales de los pacientes de sexo masculino, se incluyen micropene o pene palmeado, escroto hipoplásico y criptorquidia ^2^^,^^10^; en el sexo femenino, clítoris y labios mayores hipoplásicos. Los hallazgos menos comunes incluyen anomalías renales, luxación de la cabeza radial, anomalías vertebrales como hemivértebras y escoliosis, displasia ungueal, cardiopatía congénita, labio o paladar hendido y (raramente) retraso cognitivo.

Dada la superposición fenotípica entre la omodisplasia de tipo 2 y el síndrome de Robinow autosómico, se ha postulado que, en realidad, esto puede hacer parte de un mismo espectro clínico del síndrome de Robinow autosómico con húmeros cortos predominantes y luxación de la cabeza radial ^10^.

Cuando se inició la evaluación clínica del paciente, por su similitud, se pensó inicialmente que podría tratarse de acondroplasia -la displasia esquelética más común-, ante la presencia de la prominencia frontal y el acortamiento rizomélico de las extremidades, ambos con patrón de herencia autosómica dominante.

Se presenta un caso de omodisplasia de tipo 2, resaltando su presentación clínica, hallazgos radiológicos, causa etiopatogénica y mecanismo de acción. La secuenciación del exoma es una herramienta importante para la confirmación de esta enfermedad de difícil diagnóstico.

Después del diagnóstico, es posible hacer un adecuado seguimiento multidisciplinario que puede incluir, según el caso, evaluación por ortopedia y traumatología, cirugía maxilofacial, cirugía pediátrica, otorrinolaringología, endocrinología, psicología, diversas terapias y genética médica para el seguimiento de la evolución fenotípica, y para poder aportar un adecuado asesoramiento genético familiar pues existe un riesgo de recurrencia del 50 % en los descendientes de un individuo afectado.

## References

[1] Warren HE, Louie RJ, Friez MJ, Frías JL, Leroy JG, Spranger JW (2018). Two unrelated patients with autosomal dominant omodysplasia and FRIZZLED2 mutations. Clin Case Rep.

[2] Türkmen S, Spielmann M, Güneş N, Knaus A, Flottmann R, Mundlos S (2017). A novel de novo FZD2 mutation in a patient with autosomal dominant omodysplasia. Mol Syndromol.

[3] Gordon BL, Champaigne NL, Rogers RC, Frias JL, Leroy JG. (2014). Long-term observation of a patient with dominant omodysplasia. Am J Med Genet A.

[4] Venditti CP, Farmer J, Russell KL, Friedrich CA, Alter C, Canning D (2002). Omodysplasia: An affected mother and son. Am J Med Genet.

[5] Maroteaux P, Sauvegrain J, Chrispin A, Farriaux JP. (1989). Omodysplasia. Am J Med Genet.

[6] Nagasaki K, Nishimura G, Kikuchi T, Nyuzuki H, Sasaki S, Ogawa Y (2018). Nonsense mutations in FZD2 cause autosomal-dominant omodysplasia: Robinow syndrome-like phenotypes. Am J Med Genet A.

[7] Unger S, Ferreira CR, Mortier GR, Ali H, Bertola DR, Calder A (2023). Nosology of genetic skeletal disorders: 2023 revision. Am J Med Genet A.

[8] Saal HM, Prows CA, Guerreiro I, Donlin M, Knudson L, Sund KL (2015). A mutation in FRIZZLED2 impairs Wnt signaling and causes autosomal dominant omodysplasia. Hum Mol Genet.

[9] Arabzadeh A, Baghianimoghadam B, Nabian MH, Fallah Y, Ebrahimnasab MM. (2022). Dominant omodysplasia-A sporadic case-A new case report and review of the literature. Clin Case Rep.

[10] Roifman M, Brunner H, Lohr J, Mazzeu J, Chitayat D. (1993). Autosomal dominant Robinow syndrome.

